# Dysfunction of Self-Regulation and Self-Control in Facebook Addiction

**DOI:** 10.1007/s11126-015-9403-1

**Published:** 2015-11-20

**Authors:** Agata Błachnio, Aneta Przepiorka

**Affiliations:** Institute of Psychology, The John Paul II Catholic University of Lublin, Al. Racławickie 14, 20-950 Lublin, Poland

**Keywords:** Facebook intrusion, Self-control, Self-regulation, Multitasking, Action control

## Abstract

Nowadays, Facebook has become one of the most popular communication tools. With its increasing popularity, a new phenomenon connected with extensive use has appeared: namely, Facebook intrusion. The answer to the question of who is prone to become addicted still remains open. This study aimed to explore whether insufficient self-control and self-regulation resources in Facebook users are related to Facebook addiction. The participants in the study were 284 people. The Facebook Intrusion Questionnaire, the Brief Self-Control Scale, the Self-Regulation Scale, Action Control Scale, and the Multitasking Scale were used. We found that dysfunctional self-control system can be related to Facebook addiction. An insufficient self-control and low level of failure-related action orientation are those psychological characteristics that put Facebook users “at-risk” of Facebook addiction. The study reveals a picture showing that those Facebook users who are able to resist an impulse or temptation, are more self-disciplined, and do not focus on negative emotions are less likely to develop Facebook addiction. The obtained findings may serve as a basis for prevention programs for people at risk of Facebook addiction.

## Introduction

Facebook has become one of the popular social networking sites. Since it was launched in 2004, the number of users has reached over 1 billion [1]. Entertainment, communication, maintaining or establishing relationships, promoting oneself, and marketing—these are only a few applications of this online platform. People spend more and more time on Facebook, which is clearly visible in the statistics, where 70 % of a sample of 1597 Facebook users reported using this platform daily [2]. Additionally, given that Internet addiction is recognized worldwide as a challenge for modern society (e.g., [3], excessive Facebook use raises public concerns as well. This is mainly reflected in the rising number of publications on that subject [4, 5].

As clinicians stress, online addiction leads to a disruption of everyday life and social relationships [6]. Similar addiction symptoms and severe consequences can be caused by Facebook addiction [7]. The term “Facebook intrusion” refers to extensive engagement in Facebook [7], and in the present article it is used interchangeably with Facebook addiction. Bearing this in mind, there is an urgent need to explore this phenomenon more thoroughly in order to confirm its addiction status [8]. The present study aims to fill this gap and to examine the characteristics related to extensive Facebook usage. In the literature on Internet addiction there is some evidence that points to the role of dysfunction of self-regulation or self-control in developing Internet addiction.

Self-control is very important and beneficial to human psyche and is connected with happy and healthy life [9]. A high level of self-control is positively related to other adaptive traits such as self-esteem or good interpersonal skills, and negatively with alcohol abuse [9]. Whether people can attain goals that require self-control depends on the strength of their self-control. According to Muraven and Baumeister [10], self-control “resembles a muscle that becomes fatigued by exertion and becomes less able to function” (p. 248). In other words, self-control has limited capacity and its strength can be depleted. Some evidence indicate that a low level of self-control is related to Internet addiction [11]. For instance, in the study by Oh [12] in the group of middle school students there was a negative relationship between Internet addiction and self-control. Similarly, Lee and Shin [13] indicated possible associations between self-control and addictive consumption of avatars as a way of explaining this behavior. A recent study showed that self-control with aggression and narcissistic personality traits was negatively related to addiction to online games [14]. It is possible to predict that self-control can play an important role in Facebook addiction.

Self-regulation is defined as a proactive process in which people organize and manage their thoughts, emotions, behaviors to achieve goals [15]. Some results indicate that defective self-regulation plays a crucial role in the maintenance of Internet addiction [16]. In the generalized problematic Internet use model, self-regulation is understood also as a component of Internet addiction [17, 18].

Another construct possibly related to extensive Facebook use is action-state orientation. It is an individual difference variable connected with the volitional processes that people use to engage in goal-directed behaviors [19]. Action orientation is related to the ability to regulate emotions, thoughts, and behaviors in accordance with intentions. State orientation is connected with the inability to devote cognitive resources to task fulfillment and involves problems in initiating or maintaining action. People with this orientation have difficulties in modifying their states [19, 20]. Those who are state-oriented concentrate more on the past, present, or future; they have problems with self-control, experience more negative emotions and persistent ruminative thoughts about alternative goals.

Multitasking can be defined as the ability to do multiple tasks simultaneously or a tendency to switch between different tasks [21]. Previous results indicate that there is a relationship between being multitasking and the quality of task performance [22]. Some findings reveal that using Facebook during studying is negatively related to grade point average in school [23]. While Ophir and colleagues [24] found that people who have the ability to multitask are more susceptible to interference from irrelevant stimuli. The results suggest that Facebook use can have an impact on academic performance. If student have the multitasking ability and an initial interest for the university, this impact is positive. If not, there is no impact [25]. The question that needs to be asked is whether or not multitasking is related to Facebook addiction.

The main aim of the study was to explore how control and regulation systems are related to Facebook addiction. We posed the question of whether insufficient self-control and self-regulation in Facebook users leads to Facebook addiction. We formulated the following hypotheses:

### H1

Self-control will be negatively related to Facebook intrusion.

### H2

Self-regulation will be negatively related to Facebook intrusion.

### H3

State orientation will be positively related to Facebook intrusion.

### H4

Multitasking ability will be positively related to Facebook intrusion.

## Method

### Participants and Procedure

The sample consisted 284 individuals, of whom 83 % were women. The mean age of the participants was *M* = 22.39 (*SD* = 5.19; range: 14–67 years). Participation was anonymous and voluntary. An ad hoc sampling procedure was used to recruit participants. A link to online questionnaires was sent to Facebook users.

### Instruments

The Facebook Intrusion Questionnaire, developed by Elphinston and Noller [7], is based on behavioral addiction components and on a scale measuring phone involvement. It consists of eight items (e.g., *I have been unable to reduce my Facebook use*) measuring the relations between the tendency to Facebook involvement and eight aspects of behavioral addiction, namely: cognitive salience, behavioral salience, interpersonal conflict, conflict with other activities, euphoria, loss of control, withdrawal, as well as relapse and reinstatement. The items are rated on a 7-point Likert scale from 1 (*strongly disagree*) to 7 (*strongly agree*). Cronbach’s α was .77.

The Brief Self-Control Scale [9] was used to measure dispositional self-control. It consists of 13 items (e.g., *I am good at resisting temptation*). Cronbach’s α was .75

The Self-Regulation Scale as adapted into Polish by Łuszczyńska and Schwartzer [26] was used to measure post-intentional self-regulation in situations of goal-pursuit. The scale consists 10 items (e.g. *I can concentrate on one activity for a long time, if necessary*). Cronbach’s α was .97.

Action Control Scale (ACS-90; [27] as adapted into Polish by Marszał-Wiśniewska [28] was used to measure action control. It consists of 36 items (e.g., *When I know I must finish something soon: A. I have to push myself to get started., B. I find it easy to get it done and over with*). The scale is divided into tree subscales: action orientation subsequent to failure vs. preoccupation (AOF), prospective and decision-related action orientation vs. hesitation (AOD), and action orientation during (successful) performance of activities (intrinsic orientation) vs. volatility (AOP). Cronbach’s α was .74 for AOF, .76 for AOD, and .55 for AOP.

The Multitasking Scale [21] was used to measure the ability to perform multiple tasks simultaneously or in rapid succession. The scale was adapted into Polish using the back-translation procedure. It contains 19 items (e.g., *I frequently keep multiple programs/browsers open on my computer*). Cronbach’s α was .81.

## Results

The descriptive statistics (means and standard deviations) of all the variables and Pearson’s *r* correlations between the variables are presented in Table 1. Facebook intrusion correlated negatively with self-control, AOF, AOD, and AOP. Moreover, Facebook intrusion and intensity were related to strategies based on emotion and avoidance in stressful situations.

**Table 1:** Means, standard deviations, and correlations between variables

	*M*	*SD*	1	2	3	4	5	6
1. Facebook addiction	2.67	1.14						
2. Self-regulation	1.28	1.30	.04					
3. Multitasking	3.05	0.58	−.01	.06				
4. Self-control	3.01	0.57	−.34***	.12	.03			
5. AOF	4.54	2.80	−.27***	−.17*	.24**	.14		
6. AOD	5.31	2.94	−.30***	.18**	.18**	.50***	.04	
7. AOP	7.90	2.16	−.15*	.22***	.10	.24***	.01	.25***

AOF—action orientation subsequent to failure vs. preoccupation, AOD—prospective and decision-related action orientation vs. hesitation, AOP—action orientation during (successful) performance of activities vs. volatility

*p < .05; **p < .01; ***p < .001

Next, hierarchical multiple regression analysis was used in order to assess the impact of self-control, self-regulation, action control, and multitasking on Facebook intrusion. In the first step, self-control, self-regulation, and multitasking were entered, followed in the second step by action control. Table 2 presents the results of regression analysis for Facebook intrusion.

**Table 2:** Regression analysis results in predicting facebook intrusion

	Facebook intrusion	
	Step 1	Step 2
Self-regulation	.10	.08
Multitasking	−.02	.04
Self-control	−.37***	−.27***
AOF		−.19*
AOD		−.10
AOP		−.08
*R* ^2^	.12	.17
*R* ^2^ change	.14***	.06**

All beta weights are standardized; all *R* ^2^ values presented in the results are adjusted *R* ^2^

*p < .05; **p < .01; ***p < .001

The results indicated that the self-control variable accounts for a statistically significant proportion of the variance in Facebook intrusion (*R*^2^ = .12, *p* < .001; *F*(3, 176) = 9.451). The entry of the action control variables at Step 2 explained a statistically significant proportion of the variance in Facebook intrusion as well (*R*^2^ = .17, *p* = .009; *F*(3, 173) = 0.056). Self-control (β = −.27, *p* = .001) and AOF (β = −.19, *p* = .012) were found to have significant negative beta weights.

## Discussion

The main aim of the study was to establish whether insufficient self-control and self-regulation in Facebook users are associated with addiction to Facebook. The hypothesized associations between these variables were largely confirmed. The results indicate that self-control and action control are related to Facebook intrusion. As expected a low level of self-control and failure-related action orientation state orientation turned out to be predictors of Facebook intrusion. Neither self-regulation nor multitasking were related to Facebook addiction.

The study confirmed that insufficient self-control is a predictor of Facebook addiction. This is in accordance with the general trend presented in the subject literature, stressing that people with good self-control cope better in life, can hold their temper, do not abuse alcohol, keep secrets, and save money [9]. Also, some findings indicate that people with a high level of self-control can use the Internet in a healthy way [11]. Our results suggest that people who have insufficient self-control have problems with dysfunctional Facebook use.

In addition, our study showed that low level of failure-related action orientation is a predictor of Facebook intrusion. In general, people with prevailing state orientation experience more strongly engage in thoughts related to unpleasant situations involving failure, focus on negative emotions, cannot easily overcome these states, and have problems with the regulation of emotions, thoughts, and behaviors; moreover, they do not look for a solution [20]. Such Facebook users may escape from real world problems into virtual reality and engage in Facebook in order to improve their mood. These results found general support in other studies, where, for instance, emotional regulation was negatively related to excessive Facebook use [29]. Other studies show that Internet addicts use negative strategies of coping with stress [30]. Similarly, these pattern of stress coping may be present in Facebook addiction. Other explanation shows that those who use extensively Facebook have problems with quitting this and reducing the amount of online use.

Contrary to our expectations, self-regulation is not related to Facebook addiction. Self-regulation is a broad concept and more studies should be done to explore these associations. In Schwarzer’s theory, self-regulation refers to the situation of goal-pursuit and reflects attention regulation and emotion regulation; the specificity of the scale was not adequate in the context of Facebook use. Also, we predicted that multitasking ability would be related to Facebook addiction and that those people who have a tendency to do different activities at one time would have problems with extensive Facebook use. However, our results did not confirm these predictions.

Notwithstanding its strengths, some limitations of the study should be pointed out. First of all, cross-sectional design and self-report measurement can be here mentioned. Especially in the self-report multitasking scale the experimental approach to the examination of this ability would be more reliable. Secondly, women constituted the majority of the sample. Therefore, a closer examination of the gender effect was impossible.

To sum up, we found that dysfunctional self-control system can be related to Facebook addiction. An insufficient self-control and low level of failure-related action orientation (focus on negative emotions) are those psychological characteristics that put Facebook users “at-risk” of Facebook addiction. The study reveals a picture showing that those Facebook users who are able to resist an impulse or temptation, are more self-disciplined, and do not focus on negative emotions are less likely to develop Facebook addiction. The obtained findings may serve as a basis for prevention programs for people at risk of Facebook addiction. Nonetheless, it is necessary to continue research on dysfunctional self-control and self-regulation system in the context of Facebook addiction.
